# Dopant activation process in Mg-implanted GaN studied by monoenergetic positron beam

**DOI:** 10.1038/s41598-021-00102-2

**Published:** 2021-10-19

**Authors:** Akira Uedono, Ryo Tanaka, Shinya Takashima, Katsunori Ueno, Masaharu Edo, Kohei Shima, Kazunobu Kojima, Shigefusa F. Chichibu, Shoji Ishibashi

**Affiliations:** 1 Division of Applied Physics, Faculty of Pure and Applied Science, University of Tsukuba, Tsukuba, Ibaraki 305-8573 Japan; 2 Advanced Technology Laboratory, Fuji Electric Co., Ltd., Hino, Tokyo 191-8502 Japan; 3 Institute of Multidisciplinary Research for Advanced Materials, Tohoku University, Sendai, 980-8577 Japan; 4 Research Center for Computational Design of Advanced Functional Materials (CD-FMat), National Institute of Advanced Industrial Science and Technology (AIST), Tsukuba, Ibaraki 305-8568 Japan

**Keywords:** Electrical and electronic engineering, Materials for devices

## Abstract

A process for activating Mg and its relationship with vacancy-type defects in Mg-implanted GaN were studied by positron annihilation spectroscopy. Mg^+^ ions were implanted with an energy of 10 keV, and the Mg concentration in the subsurface region (≤ 50 nm) was on the order of 10^19^ cm^−3^. After the Mg-implantation, N^+^ ions were implanted to provide a 300-nm-deep box profile with a N concentration of 6 × 10^18^ cm^−3^. From capacitance–voltage measurements, the sequential implantation of N was found to enhance the activation of Mg. For N-implanted GaN before annealing, the major defect species were determined to Ga-vacancy related defects such as divacancy. After annealing below 1000 °C, the clustering of vacancies was observed. Above 1200 °C annealing, however, the size of the vacancies started to decrease, which was due to recombinations of vacancy clusters and excess N atoms in the damaged region. The suppression of vacancy clustering by sequential N-implantation in Mg-implanted GaN was attributed to the origin of the enhancement of the Mg activation.

## Introduction

Gallium nitride (GaN) is a promising material candidate for next generation power electronics^1–3^. Because of its wide bandgap, high saturation electron velocity, sufficient thermal conductivity, and high breakdown voltage, GaN yields a higher Baliga’s figure of merit compared with that of semiconductors for power devices such as Si and SiC^4^. Depending on the structures of GaN devices, they can be categorized into lateral and vertical devices^5–7^. For lateral GaN devices, a two-dimensional electron gas at the AlGaN/GaN interface gives electrons high mobility in channel and drain drift regions. These GaN devices have been developed for high-frequency power switching applications for ratings of up to a few kW. Using this structure, however, the chip size of transistors tends to be increased in order to increase power ratings, and this causes difficulty in current extraction^2^. Because of carrier trapping by interface states between gate dielectrics and GaN or AlGaN layers, the maximum output power of the lateral devices is limited, which results in the suppression of drain currents^8,9^. Although several methods for reducing this current collapse effect exist^1^, such as surface passivation and field plate structures, the phenomenon is one of the major problems that degrades electric properties and the reliability of the lateral devices. Vertical GaN devices offer inherent advantages such as the capability of achieving a high breakdown voltage by increasing the thickness of the drift region without enlarging the chip size^2,3,6,10^. The peak electric field in the vertical devices is away from the surface, which minimizes carrier trapping effects, reduces dynamic on-resistance, and makes thermal management easier compared with the lateral devices.

In Si- and SiC power devices, their high breakdown voltage is achieved by edge termination technology such as the formation of junction termination extension structures and field rings. These structures are formed by selective p-type doping using ion implantation. For vertical GaN devices, this technique must be also effective. Because the threshold voltage of the devices is determined by the net acceptor concentration (*N*_a_) in p-type body layers, the precise control of *N*_a_ is crucial, where the depth distribution of *N*_a_ should be uniform to avoid punch-through under a high drain bias. Mg concentrations in p-type bodies and contact layers are required to be on the order of 10^17^ and 10^20^ cm^−3^, respectively^10^. Ion implantation is the most suitable technique for fabricating these layers with such a wide doping range in a limited area. In conclusion, acceptor (Mg) ion implantation is indispensable for fabricating vertical GaN devices.

The activation of implanted dopants, however, is not easy for GaN^11^. Ion implantation introduces point defects, such as vacancies and interstitials, through energetic collision cascades of ions. The damage could be recovered by annealing, but the defects interact with each other and could form permanent complexes or clusters. These defects could act as compensators of p-type dopant and/or be an origin causing the deactivation of Mg. Several annealing processes have been developed to overcome these obstacles. Feigelson et al.^12^ successfully used multicycle rapid thermal annealing (MRTA) to activate implanted Mg in GaN. The p-type conductivity of Mg-implanted GaN was confirmed by using Hall measurements, where the activation efficiency was over 8%. They also reported that the crystallinity of GaN can be further improved by using additional post-pulse annealing in the MRTA process^13^. Recently, the ultra-high-pressure annealing (UHPA) process was applied to Mg-implanted GaN, and it was confirmed that high-temperature annealing (1300–1480 °C) under ultra-high N_2_ pressure (1 GPa) was effective at activating implanted Mg in GaN^10,14,15^. These works reported that the activation rate of Mg exceeded 70%, and the carrier mobility was close to that of epitaxial p-type GaN with the same doping concentration. Although these results suggest that the UHPA process is a promising post-implantation process, several technical issues, such as the inclusion of impurities, costs, throughput, etc., must be overcome for industrial applications of UHPA. Thus, a subsequent ion implantation (or co-implantation) technique that is used before annealing at atmospheric pressure is also a potential candidate^16,17^.

Another technical barrier to the utilization of ion implantation is the deformation of dopant depth profiles during annealing. The diffusion of Mg starts at the temperature required for its activation (≥ 1300 °C)^10,16–18^. During the annealing process, Mg atoms diffuse in damaged regions introduced by ion implantation, and they interact with various defects such as vacancies and interstitials. Thus, knowledge on interactions between Mg and point defects is key to controlling not only the activation of Mg but also its depth profile. In the present study, we implanted N atoms after the implantation of Mg, and vacancies introduced by N-implantation were used to suppress the diffusion of Mg during annealing. Annealing behaviors of implantation induced vacancies were studied by using positron annihilation spectroscopy, and the relationship between the activation of Mg and vacancies was discussed.

## Experimental

### Mg and N-implantation into GaN

The GaN samples used in the present experiment were 4-µm-thick undoped GaN layers deposited on GaN substrates by using metal–organic vapor phase deposition. The GaN substrates in the *c* + -direction were grown by using hydride vapor phase epitaxy. The dislocation density in the substrates was estimated to be lower than 10^7^ cm^−2^. Mg^+^ ions were implanted with an implantation energy of 10 keV and a dose of 9 × 10^13^ cm^−2^, where the Mg concentrations ([Mg]) in the subsurface region (≤ 50 nm) were on the order of 10^19^ cm^−3^. After the Mg-implantation, N^+^ ions were implanted with energies of 15–180 keV in order to obtain a 300-nm-deep box profile with a N concentration of 6 × 10^18^ cm^−3^. After the ion implantation, 300-nm-thick AlN films were deposited on the samples by using a sputtering technique to encapsulate the surface. The samples were annealed at temperatures from 1000 to 1300 °C (5 min) in flowing N_2_ gas at atmospheric pressure. After annealing, the AlN cap was removed by KOH based selective wet chemical etching^19^.

The depth distributions of Mg in the samples before and after 1300 °C were measured by secondary ion mass spectrometry (SIMS). The depth profiles of Mg and N for as-implanted GaN were calculated by using SRIM code^20^. The depth distributions of *N*_a_ were estimated by using a capacitance–voltage (*C*–*V*) profiling technique^21^, where the value of *N*_a_ was obtained from the slope of the relationship between 1/*C*^2^ and *V*. In this technique, the depth of a sample equals the depletion layer width *w*_d_, where *w*_d_ can be estimated by using the junction area and *C*. A Ni/Au Schottky contact (Φ400 μm) was deposited on the Mg-implanted GaN layer by using an electron beam evaporation, and Ti/Al ohmic contacts were deposited on the GaN substrate. The *C*–*V* characteristics were measured at frequencies of 1 kHz by using a Keysight E4980A LCR meter.

### Positron annihilation spectroscopy

Details on positron annihilation spectroscopy^22,23^ and its application to Mg-implanted GaN are given elsewhere^24–26^. Using a monoenergetic positron beam, the Doppler broadening spectra of the annihilation radiation were measured as a function of the incident positron energy *E* by using two Ge detectors. The spectra were evaluated by the *S* parameter, defined as the fraction of annihilation events in the energy range of 510.22‒511.78 keV, and by the *W* parameter, defined as the number of events in the ranges of 504.14‒507.96 keV and 514.04‒517.86 keV. The energy resolution of the Ge detectors was 1.2–1.3 keV (full-width at half-maximum: FWHM). Doppler broadening profiles were also measured with a coincidence system^22,23^ in darkness and while the samples were illuminated with a He-Cd laser (wavelength: 325 nm). Depth distributions of *S* were obtained from an analysis of *S*–*E* curves by using VEPFIT code^27^. The Doppler broadening spectra were calculated by using QMAS (Quantum MAterials Simulator) code^28,29^. The exchange and correlation energy of electrons was described by generalized gradient approximation^30^. The positron wave function was calculated by using the formalism of the local density approximation^31^. Orthorhombic supercells equivalent to 4 × 4 × 2 wurtzite cells containing 128 atoms were used for calculation (if there were no vacancies in a cell). For supercells containing a defect, atomic positions in the cells were optimized. The calculated spectra were convoluted with the energy resolution of the coincidence system (0.9 keV).

## Results and discussion

### Activation of implanted Mg in GaN by annealing at 1300 °C

**Figure 1:**
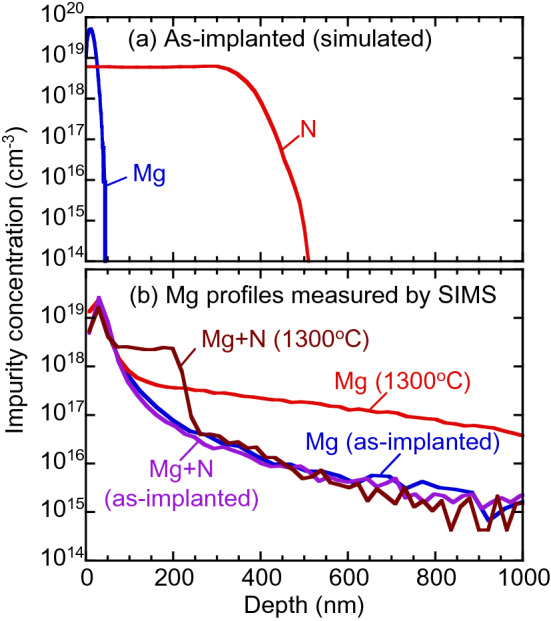
(**a**) Computer simulated depth distributions of Mg and N for as-implanted GaN. (**b**) Depth distributions of Mg for Mg-implanted GaN with and without N-implantation (denoted as “Mg + N” and “Mg”) measured by SIMS. Annealing temperature is shown in figure.

Figure 1 shows (a) the calculated depth distributions of Mg and N for as-implanted GaN, and (b) the distributions for Mg-implanted GaN before and after annealing at 1300 °C. The blue line (denoted as “Mg + N”) shows the Mg profile for the sample with N-implantation before annealing. From a comparison between the calculated and experimentally obtained profiles of Mg in as-implanted GaN, the observed long tail in the region below 100 nm can be attributed to the implantation of Mg by channeling. No large change in the Mg profile was observed below 1200 °C annealing (not shown), but Mg started to diffuse into the bulk after annealing at 1300 °C. For the sample without N-implantation (red line), [Mg] reached 4 × 10^16^ cm^−3^ at a depth of 1 μm. For the sample with N-implantation (brown line), after annealing at the same temperature, the diffusion of Mg was suppressed, and [Mg] was almost constant in the region between 50 and 200 nm. The observed box-like profile of Mg is unlikely to be reproducible by a diffusion equation without assuming interactions between Mg and defects. We discuss this later.

**Figure 2:**
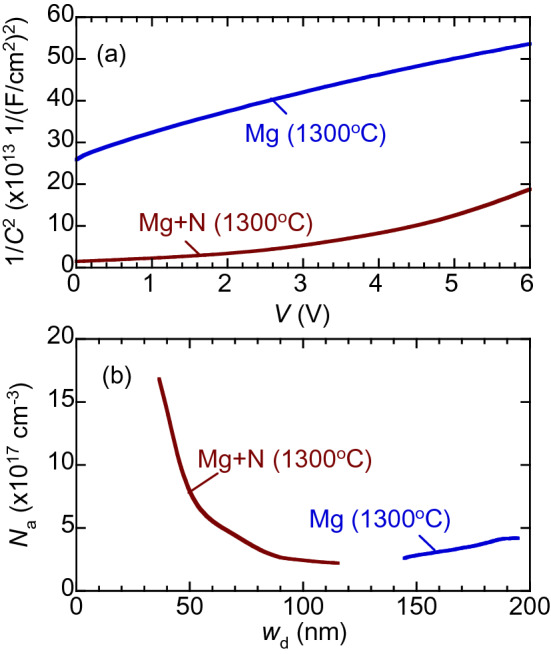
(**a**) 1/*C*^2^ versus *V* for Mg-implanted GaN with and without N-implantation. (**b**) Relationships between net acceptor density *N*_a_ and width of depletion layer *w*_d_.

Figure 2 shows (a) the relationship between 1/*C*^2^ and *V* and (b) the depth distributions of *N*_a_ for Mg-implanted GaN with and without N-implantation after annealing at 1300 °C. For the sample with N-implantation, a certain amount of Mg was activated in the subsurface region (≤ 50 nm), which corresponds to the region with high [Mg] (Fig. 1). For the sample without N-implantation, this was not the case. The obtained results suggest that the sequential implantation of N into Mg-implanted GaN enhanced the activation of the implanted Mg.

### Annealing behaviors of vacancies in Mg-implanted GaN

**Figure 3:**
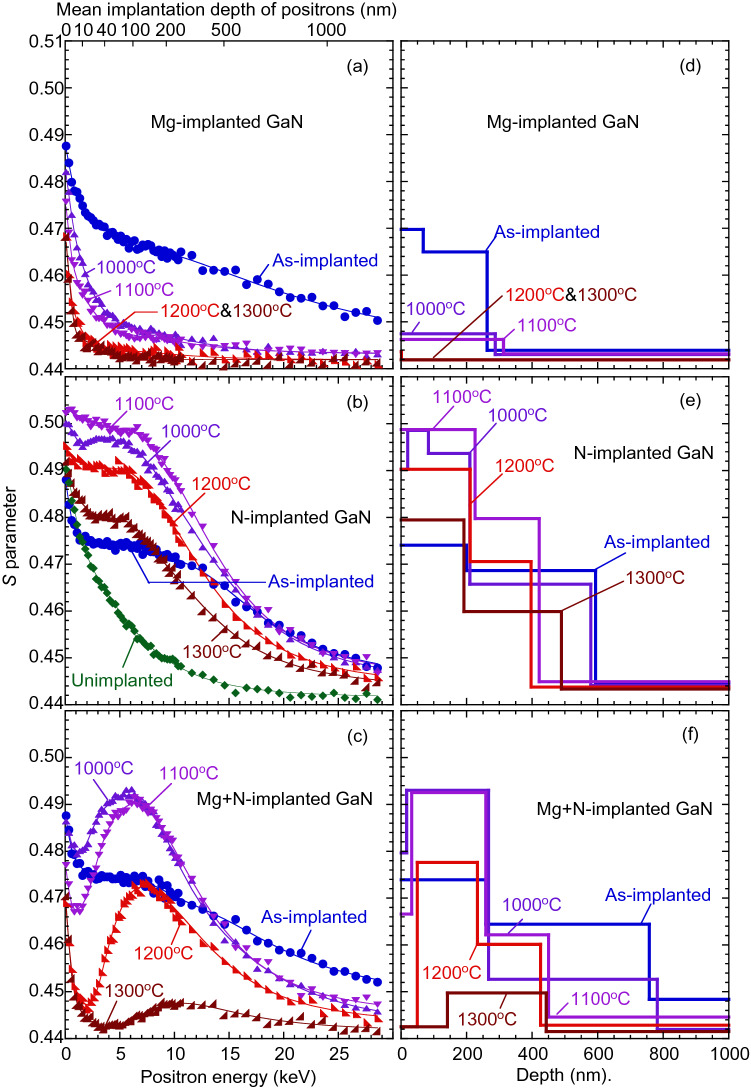
*S* parameters as function of incident positron energy *E* for (**a**) Mg-implanted GaN, (**b**) N-implanted GaN, and (**c**) Mg-implanted GaN with N-implantation. *S*–*E* curve for unimplanted GaN is shown in (**b**). Annealing temperatures (1000–1300 °C) are shown in figure. Depth distributions of *S* obtained from analysis of *S*–*E* curves shown in (**d**), (**e**), and (**f**), respectively.

Figure 3 shows the *S* values of (a) Mg-implanted GaN, (b) N-implanted GaN, and (c) Mg-implanted GaN with N-implantation. All *S*–*E* curves were measured in darkness. The mean positron implantation depth is shown on the upper horizontal axis of Fig. 3a. The result for unimplanted GaN was also shown in Fig. 3b. For unimplanted GaN, the *S* value at a low *E* (0.1 keV) was high, which was due to the annihilation of positrons at the surface. The *S* value saturated above *E* = 20 keV, which is due to the annihilation of positrons from the delocalized state in GaN^24–26^. Thus, the shoulders in the *S*–*E* curves for ion implanted GaN before annealing (*E* = 3–10 keV) can be attributed to the trapping of positrons by vacancy-type defects introduced by ion implantation. For Mg-implanted GaN without N-implantation, the *S* values were decreased after annealing. For the samples annealed at 1200 and 1300 °C, the *S* values at *E* > 5 keV were close to *S* for undoped GaN with *E* ≥ 20 keV, suggesting that positrons mainly annihilated from the delocalized state in these samples.

The solid curves in Fig. 3a–c are fits to the experimental data. For unimplanted GaN, a homogeneous distribution of *S* was assumed, and the diffusion length of positrons (*L*_d_) was determined to be 119 ± 2 nm. For ion implanted GaN, the region sampled by positrons was divided into several blocks. Their locations were determined by the fitting, where the minimum number of blocks was used to obtain appropriate fitting results. The block located in the deepest region corresponds to the defect-free region, and the value of *L*_d_ was fixed as the one obtained for unimplanted GaN. The values of *L*_d_ in the blocks closest to the surface were determined by the fitting, and they were obtained to be 5–10 nm. The values of *L*_d_ for the other blocks (except the deepest one) were fixed as *L*_d_ obtained for the first block. The short positron diffusion length in the damaged region suggests that almost all positrons annihilated from the trapped state due to vacancies.

Figure 3d shows the derived depth distributions of *S* for the Mg-implanted GaN. For the as-implanted sample, the *S* value in the subsurface region (< 50 nm) was higher than that in the deeper damaged region (50–250 nm). This subsurface region was close to the mean implantation depth of Mg (Fig. 1), and the introduction of vacancies at the depth of 50–250 nm can be attributed to the channeling of Mg. Figure 3b shows the *S*–*E* curves for N-implanted GaN, and the derived depth distributions of *S* are shown in Fig. 3e. As shown in Fig. 3b, e, the *S* value was increased by the annealing, which can be attributed to the introduction of vacancy clusters. In Fig. 3e, a region with a high *S* value was introduced in the region of 0–200 nm after annealing at 1000°C, suggesting an increase in the size of vacancy-type defects. A similar distribution of *S* was observed even after 1300 °C annealing, and the region with a high *S* agreed with the box-like profile of Mg in Mg-implanted GaN with N-implantation (Fig. 1). Thus, it can be concluded that the diffusion of Mg was suppressed by vacancy-type defects introduced by N-implantation, and Mg atoms tended to accumulate in the region with a high concentration of vacancy-type defects during annealing at 1300°C. This fact suggests that the depth profile of dopants after high-temperature annealing can be controlled by vacancy-type defects introduced by sequential implantation (or co-implantation).

The trapping property of positrons by vacancy-type defects in Mg-implanted GaN was reported in previous works^24–26^. Annealing behaviors of vacancy-type defects for Mg-implanted GaN with [Mg] = 10^17^–10^19^ cm^−3^ were reported in ref.^24^. Although the concentration of residual vacancies after annealing was expected to increase with increasing [Mg]^11^, the *S* value in the damaged region for the sample with [Mg] = 10^17^ cm^−3^ was higher than that for the samples with [Mg] = 10^18^ and 10^19^ cm^−3^, suggesting that the trapping rate of positron by vacancies decreased with increasing [Mg]. The trapping rate of positrons by positively charged vacancies is negligible compared with that by neutral or negatively charged vacancies^22,23^. As the annealing temperature increases, Mg is partially activated, and as a result, the Fermi level position approaches the valence band maximum (VBM). For example, Lyons and Van de Walle calculated the thermodynamic transition levels of point defects in GaN and reported that the charge state of Ga-vacancy (*V*_Ga_) is positive when the Fermi level position locates at about 1 eV above VBM^32^. A similar relationship between the defect charge states of *V*_Ga_-related defects and the Fermi level position can be expected^33^. The defect charge states in Mg-implanted GaN tend to be positive with an increased annealing temperature, and this causes the decrease in the trapping rate of positrons by such defects. Thus, the observed decrease in *S* for Mg-implanted GaN (Fig. 3a, d) is unlikely due to the annealing out of vacancy-type defects but the downward shift of the Fermi level position and a resultant increase in positively charged vacancy-type defects.

Figure 3c, f show the *S*–*E* curves and the depth distributions of *S* for Mg-implanted GaN with N-implantation. Before annealing, no large difference in the *S*–*E* curves for the Mg-implanted sample with N-implantation and the N-implanted sample was observed (Fig. 3b, c). After annealing at 1000°C, although the *S* value at *E* = 5 keV increased, the change in *S* at *E* = 1–2 keV was suppressed. Above 1200°C annealing, the decrease in *S* at a low *E* and the annealing behavior of *S* at *E* > 3 keV can be attributed to the partial activation of Mg in the subsurface region and vacancy clustering in the deeper region. In Fig. 3f, for the sample annealed at 1300°C, the *S* value at the subsurface region (0–150 nm) was close to the defect-free *S* value, which can be attributed to the activation of Mg in the subsurface region (see Fig. 2).

**Figure 4:**
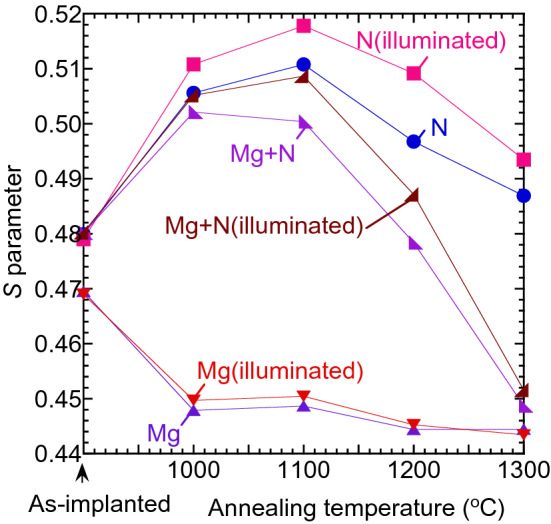
Annealing behaviors of *S* for Mg- and N-implanted GaN. Result for Mg-implanted GaN with N-implantation is also shown. *S* values were measured by using coincidence system in darkness and under illumination.

Figure 4 shows the annealing behaviors of *S* calculated from coincidence Doppler broadening spectra, where the measurements were done in darkness and under illumination. For Mg-implanted GaN and N-implanted GaN, the *S* values were measured at *E* = 8.1 keV and 4.1 keV, respectively. For Mg-implanted GaN with N-implantation before and after annealing at 1000, 1100, 1200, and 1300°C, the *S* values were measured at 4.1, 6.1, 6.1, 7.6, and 10.1 keV, respectively, where the mean implantation depth of positrons with *E* = 6–10 keV ranged between 100 and 200 nm. These energies were chosen to follow the largest *S* value in the *S*–*E* curve at each annealing temperature (see Fig. 3). For N-implanted GaN, the *S* value measured in darkness increased as the annealing temperature increased, and it started to decrease above 1200°C annealing. The observed change in *S* can be attributed to the agglomeration and dissociation of vacancy-type defects during annealing. For Mg-implanted GaN with N-implantation, the *S* values were smaller than those for N-implanted GaN. This can be attributed to the activation of Mg implanted in the region of 100–200 nm.

**Figure 5:**
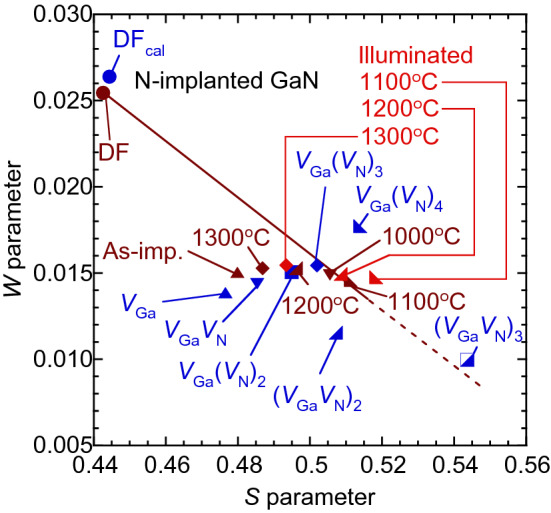
*S*–*W* relationship for N-implanted GaN measured in darkness (brown symbols) and under illumination (pink), where annealing temperatures are shown in figure. (*S*,*W*) for unimplanted GaN is shown as “DF.” Calculated values for positron annihilation in defect-free GaN (DF_cal_), *V*_Ga_, *V*_Ga_(*V*_N_)_n_ (n = 1–4), (*V*_Ga_*V*_N_)_2_, and (*V*_Ga_*V*_N_)_3_ are also shown (blue symbols).

The defect species detected by positron annihilation can be identified by using the relationship between the *S* and *W* values^22,23^. Figure 5 shows the *S*–*W* relationship for N-implanted GaN before and after annealing, where the values were obtained from the coincidence Doppler broadening spectrum in darkness (brown symbols) and under illumination (pink symbols). These values were measured at *E* = 4.1 keV. The statistical error of the (*S*,*W*) values was close to the size of the symbol used in the figure. The (*S*,*W*) value corresponding to the positron annihilation in the delocalized state is shown as “DF.” The calculated (*S*,*W*) values for the annihilation of positrons in the delocalized state (DF_cal_) as well as typical cation vacancies, such as *V*_Ga_, and complexes between *V*_Ga_ and N vacancy (*V*_N_) [*V*_Ga_(*V*_N_)_n_ (n = 1–3), (*V*_Ga_*V*_N_)_2_, and (*V*_Ga_*V*_N_)_3_], are also shown in Fig. 5 (blue symbols). For the as-implanted sample (denoted as “As-imp.”), the observed (*S*,*W*) value was close to the calculated value for *V*_Ga_-related defects such as *V*_Ga_*V*_N_. Thus, the major defect species for the sample before annealing can be identified as such defects. Since the (*S*,*W*) value for Mg-implanted GaN with N-implantation was identical to that for N-implanted GaN (not shown), the same identification of the defect species can be done. This conclusion agrees with that obtained for Mg-implanted GaN with different implantation energies and implantation dosages^24–26,34^.

After annealing at 1000 and 1100°C, the (*S*,*W*) shifted toward the right-hand side. The calculated (*S*,*W*) value for (*V*_Ga_*V*_N_)_3_ was close to the extended line connecting the values for the unimplanted sample and the sample annealed at these temperatures (dotted line). Similar annealing behaviors of (*S*,*W*) were reported for Mg-implanted GaN with a 500-nm-deep box profile with [Mg] = 10^17^–10^19^ cm^−3^ ^24^. In Ref.^24^, from measurements of positron lifetime spectra, the major defect species were identified to be vacancy-clusters such as (*V*_Ga_*V*_N_)_3_. Thus, similar vacancy-clusters were considered to be introduced for N-implanted GaN after annealing at 1000–1100 °C. In Ref.^24^, further annealing caused a shift of (*S*,*W*) toward the value of DF along this line. For N-implanted GaN, however, the value tended to return back to the value for the as-implanted sample. This behavior can be attributed to a decrease in the size of vacancy clusters. An excess number of N in the damaged region is considered to suppress the increase in the size of vacancies. For Mg-implanted GaN with N-implantation, the same suppression of vacancy clustering in the region with high [Mg] is expected, and this is an origin for the enhancement of Mg activation due to sequential N-implantation.

Figure 4 shows the *S* values measured under illumination. For N-implanted GaN after annealing, the *S* value increased with the illumination. For Mg-implanted GaN and GaN grown on Si, an increase in *S* under illumination was reported^24–26,35^, and this was attributed to the trapping of electrons excited by illumination and a resultant transition in the charge state of vacancies (*V*) from positive to neutral (or neutral to negative) *V* ^+^ → *V*^0^ (or *V* ^0^ → *V*^*−*^). For N-implanted GaN, the location of the Fermi level position is supposed to be close to the center of the band gap. Thus, the observed increase in *S* suggests that a certain number of vacancies with an energy level of +/0 (or 0/−) above the center of the band gap were formed after annealing. In Fig. 5, the (*S*,*W*) value tended to shift toward the right-hand side under illumination, suggesting that the size of the vacancies responding to the illumination was larger than that of the vacancies detected in darkness. The same trend of *S* for Mg-implanted GaN with N-implantation was observed. For Mg-implanted GaN, the effect of the illumination was small. The suppression of illumination effect on *S* was observed for Mg-implanted GaN with high [Mg]^24^ and GaN films with high [C]^35^. These p-type impurities doped with high concentration are supposed to trap electrons excited by illumination. Thus, the observed small illumination effect can be attributed to the trapping of excited electrons by Mg and a resultant suppression of their trapping by vacancy-type defects.

## Summary

We used positron annihilation to study the enhancement of Mg activation and its relationship to vacancy-type defects in Mg-implanted GaN with N-implantation. For an as-implanted sample, the major defect species were identified to be *V*_Ga_-related defects. For N-implanted GaN, the clustering of vacancies was observed after annealing at 1000 and 1100 °C. After annealing above 1200 °C, however, the size of the vacancies was observed to shrink, which was due to recombinations between vacancy clusters and excess N in the damaged region. From *C*–*V* measurements for Mg-implanted GaN with N-implantation, the activation of Mg in the subsurface region (≤ 50 nm) was observed, but for the sample without N-implantation, this was not the case. The observed enhancement of Mg activation was attributed to the suppression of vacancy clustering by sequential N-implantation. For Mg-implanted GaN without N-implantation, the diffusion of Mg toward the bulk started after annealing at 1300 °C. For the sample with N-implantation, the diffusion of Mg was suppressed, and a 200-nm box-like profile of Mg was formed, which was attributed to the trapping of Mg by vacancies introduced by N-implantation. The observed phenomenon suggests that sequential ion-implantation can be used to modify and control the dopant profile, and this is effective at forming shallow depth distributions of Mg for p-contact formation.

## References

[1] Amano H (2018). The 2018 GaN power electronics roadmap. J. Phys. D: Appl. Phys..

[2] Hu J, Zhang Y, Sun M, Piedra D, Chowdhury N, Palacios T (2018). Materials and processing issues in vertical GaN power electronics. Mat. Sci. Semicond. Process..

[3] Oka T (2019). Recent development of vertical GaN power devices. Jpn. J. Appl. Phys..

[4] Baliga BJ (2013). Gallium nitride devices for power electronic applications. Semicond. Sci. Technol..

[5] Fletcher ASA, Nirmal D (2017). A survey of gallium nitride HEMT for RF and high power applications. Superlattices Microstruct..

[6] Zhang YH, Dadgar A, Palacios T (2018). Gallium nitride vertical power devices on foreign substrates: A review and outlook. J. Phys. D: Appl. Phys..

[7] Greco G, Iucolano F, Roccaforte F (2018). Review of technology for normally-off HEMTs with p-GaN gate. Mat. Sci. Semicond. Process..

[8] Hu X, Koudymov A, Simin G, Yang J, Khan MA, Tarakji A, Shur MS, Gaska R (2001). Si_3_N_4_/AlGaN/GaN-metal-insulator-semiconductor heterostructure field-effect transistors. Appl. Phys. Lett..

[9] Klein PB, Binari SC, Ikossi K, Wickenden AE, Koleske DD, Henry RL (2001). Current collapse and the role of carbon in AlGaN/GaN high electron mobility transistors grown by metalorganic vapor-phase epitaxy. Appl. Phys. Lett..

[10] Narita T, Yoshida H, Tomita K, Kataoka K, Sakurai H, Horita M, Bockowski M, Ikarashi N, Suda J, Kachi T, Tokuda Y (2020). Progress on and challenges of p-type formation for GaN power devices. J. Appl. Phys..

[11] Kucheyev SO, Williams JS, Pearton SJ (2001). Ion implantation into GaN. Mat. Sci. Eng..

[12] Feigelson BN, Anderson TJ, Abraham M, Freitas JA, Hite JK, Eddy CR, Kub FJ (2012). Multicycle rapid thermal annealing technique and its application for the electrical activation of Mg implanted in GaN. J. Cryst. Growth.

[13] Greenlee JD, Anderson TJ, Feigelson BN, Hobart KD, Kub FJ (2015). Characterization of an Mg-implanted GaN p-i-n diode. Phys. Stat. Sol. A.

[14] Sakurai H, Omori M, Yamada S, Furukawa Y, Suzuki H, Narita T, Kataoka K, Horita M, Bockowski M, Suda J, Kachi T (2019). Highly effective activation of Mg-implanted p-type GaN by ultra-high-pressure annealing. Appl. Phys. Lett..

[15] Matys M, Ishida T, Nam KP, Sakurai H, Narita T, Uesugi T, Bockowski M, Suda J, Kachi T (2021). Mg-implanted bevel edge termination structure for GaN power device applications. Appl. Phys. Lett..

[16] Narita T, Sakurai H, Bockowski M, Kataoka K, Suda J, Kachi T (2019). Electric-field-induced simultaneous diffusion of Mg and H in Mg-doped GaN prepared using ultra-high-pressure annealing. Appl. Phys. Exp..

[17] Tanaka R, Takashima S, Ueno K, Matsuyama H, Edo M (2020). Demonstration of 1200 V/1.4 mΩcm^2^ vertical GaN planar MOSFET fabricated by an all ion implantation process. Jpn. J. Appl. Phys..

[18] Sakurai H, Narita T, Omori M, Yamada S, Koura A, Iwinska M, Kataoka K, Horita M, Ikarashi N, Bockowski M, Suda J, Kachi T (2020). Redistribution of Mg and H atoms in Mg-implanted GaN through ultra-high-pressure annealing. Appl. Phys. Exp..

[19] Guo W, Kirste R, Bryan I, Bryan Z, Hussey L, Reddy P, Tweedie J, Collazo R, Sitar Z (2015). KOH based selective wet chemical etching of AlN, Al_x_Ga_1−x_N, and GaN crystals: A way towards substrate removal in deep ultraviolet-light emitting diode. Appl. Phys. Lett..

[20] Ziegler JF, Ziegler MD, Biersack JP (2010). SRIM—The stopping and range of ions in matter. Nucl. Instrum. Methods B.

[21] Sze SM, Ng KK (2007). Physics of Semiconductor Devices.

[22] Krause-Rehberg R, Leipner HS (1999). Positron Annihilation in Semiconductors, Solid-State Sciences.

[23] Tuomisto F, Makkonen I (2013). Defect identification in semiconductors with positron annihilation: Experiment and theory. Rev. Mod. Phys..

[24] Uedono A, Takashima S, Edo M, Ueno K, Matsuyama H, Egger W, Koschine T, Hugenschmidt C, Dickmann M, Kojima K, Chichibu SF, Ishibashi S (2018). Carrier trapping by vacancy-type defects in Mg-implanted GaN studied using monoenergetic positron beams. Phys. Stat. Sol. B.

[25] Uedono A, Iguchi H, Narita T, Kataoka K, Egger W, Koschine T, Hugenschmidt C, Dickmann M, Shima K, Kojima K, Chichibu SF, Ishibashi S (2019). Annealing behavior of vacancy-type defects in Mg- and H-implanted GaN studied using monoenergetic positron beams. Phys. Stat. Sol. B.

[26] Uedono A, Sakurai H, Narita T, Sierakowski K, Bockowski M, Suda J, Ishibashi S, Chichibu SF, Kachi T (2020). Effects of ultra-high-pressure annealing on characteristics of vacancies in Mg-implanted GaN studied using a monoenergetic positron beam. Sci. Rep..

[27] Van Veen A, Schut H, Clement M, de Nijs JMM, Kruseman A, Ijpma MR (1995). VEPFIT applied to depth profiling problems. Appl. Surf. Sci..

[28] Ishibashi S, Tamura T, Tanaka S, Kohyama M, Terakura K (2007). *Ab initio* calculations of electric-field-induced stress profiles for diamond/c−BN(110) superlattices. Phys. Rev. B.

[29] Ishibashi S, Uedono A, Kino H, Miyake T, Terakura K (2019). Computational study of positron annihilation parameters for cation mono-vacancies and vacancy complexes in nitride semiconductor alloys. J. Phys.: Condens. Matter.

[30] Perdew JP, Burke K, Ernzerhof M (1996). Generalized gradient approximation made simple. Phys. Rev. Lett..

[31] Boronski E, Nieminen RM (1986). Electron-positron density-functional theory. Phys. Rev. B.

[32] Lyons JL, Van de Walle CG (2017). Computationally predicted energies and properties of defects in GaN. NPJ Comput. Mater..

[33] Diallo IC, Demchenko DO (2016). Native point defects in GaN: A hybrid-functional study. Phys. Rev. Appl..

[34] Akazawa M, Kamoshida R, Murai S, Kachi T, Uedono A (2021). Low-temperature annealing behavior of defects in Mg-ion-implanted GaN studied using MOS diodes and monoenergetic positron beam. Jpn. J. Appl. Phys..

[35] Uedono A, Tanaka T, Ito N, Nakahara K, Egger W, Hugenschmidt C, Ishibashi S, Sumiya M (2017). Electron capture by vacancy-type defects in carbon-doped GaN studied using monoenergetic positron beams. Thin Solid Films.

